# Maternal autonomy and birth registration in India: Who gets counted?

**DOI:** 10.1371/journal.pone.0194095

**Published:** 2018-03-13

**Authors:** Itismita Mohanty, Tesfaye Alemayehu Gebremedhin

**Affiliations:** 1 Centre for Research and Action in Public Health (CeRAPH), Health Research Institute, University of Canberra, Canberra, Australia; 2 Faculty of Business, Government and Law, University of Canberra, Canberra, Australia; TNO, NETHERLANDS

## Abstract

This paper examines the effect of maternal socio-economic status in the household, such as their autonomy, ability, freedom and bargaining power, on child birth registration in India using the nationally representative India Human Development Survey-II (IHDS-II), 2011–12. We have estimated a multilevel mixed effects model which accounts for the hierarchical structure of the data and allows us to examine the effects of unobserved ‘district’ and ‘community’ characteristics along with individual child level characteristics on probability of birth registration. The results show that between-districts and between individuals differences share a considerably high and an almost equal proportion of the variations in probability of birth registration in India. At individual child level, mother’s socio-economic status such as her ability to move around independently and her exposure to outside world, significantly raise the probability of birth registration. More importantly, the marginal effects of the maternal autonomy indicators: mother’s ability to move around freely and her control over resources, on birth registration vary across districts in India. Other variables such as institutional birth, mother’s antenatal care seeking behaviour, caste, religion, household wealth and parental education are significant determinants of birth registration.

## 1. Introduction

Civil Registration and Vital Statistics (CRVS) system have renewed momentum in the new Sustainable Development Goals (SDG) guidelines. CRVS is both a target in its own right and fundamental for maternal and child health, social inclusion, access to education and health services [1]. Civil registration is the way by which countries keep a continuous and complete record of births and deaths. It is important at a national and state level for policy and planning purposes. World Health Organisation (WHO) considers it as the most reliable source of statistics. It is the birth right of each child to be registered and issued with a birth certificate [2]. Globally 35% of births go unaccounted for in registration [3]. The issue has been highlighted as *a scandal of invisibility w*here *m*ost people born in Africa and Asia die without leaving a trace in any legal record or official statistic [4]. Over the past decade there has been a significant shift in the literature from *Who Counts*? [4–7]*…*… to *Counting Births and Deaths* [3, 8–10] that established the CRVS system as a necessary component of SDG.

Globally 230 million children under the age of five have never been recorded. More than half (59%) live in Asia, and an estimated 71 million–one in three, live in India. Many barriers prevent people from registering births and deaths. There are countries that do not have the necessary system in place to make births and deaths registration mandatory whereas in other countries only urban people have access to registration services. India has started its own CRVS improvement initiatives and introduced the requisite legislative and administrative reforms to improve civil registration. As a result, birth registration coverage increased from 60% in 2001 to more than 80% in 2010 but the process is still incomplete. Over the last two decades, there has been significant emphasis on promoting access to Maternal and Child Health (MCH) services in India while similar emphasis on completing birth registration has been lacking. In the SDG era, where the goal is to promote access and equitable health for all through Universal Health Coverage (UHC), universal birth registration needs to be prioritised. Fagernas and Odame (2013) [11] note that birth registration systems would be useful in tracking progress towards health-related goals.

There is little empirical research so far to identify at individual birth and death registration level—what exactly hinders the registration process in India. This paper is an important first step that examines the individual, household, community and district level determinants of birth registration using a multilevel hierarchical mixed model. In India, the process of birth registration is based on informant reporting structure, where the primary responsibility lies on individual informants who report birth. It can be the head of the household in case of home events and institutional heads in case of institutional deliveries. Under such circumstances, where only 13% (84%) of pregnant women in the poorest (the richest) population quintile delivered in health facilities in 2005 [12, 13], it is important to focus on the relative significance of individual, household, community and district level enabling factors in determining the registration of a birth.

This paper analyses ‘if independent and informed mothers are more likely to register their children in India’. Many mothers lack the knowledge on how to register a child’s birth [2] and consequently are unaware of what it entails and delivers to their child. The status of birth registration would significantly improve across the world if women were educated, well informed and independent given their role as primary caregivers for children. However, women continue to have little household decision-making authority in many developing countries including India. Improvements in maternal socio-economic status have been strongly linked in the literature to better educational and demographic outcomes, improved child welfare and allocation of household resources in favour of children’ [14–18]. UNICEF (2013) [2] notes that mothers with some schooling are more likely to know how to register a child than their uneducated peers. In India, birth registration levels increase with mothers’ education.

## 2. Methods

### 2.1 Study design and data source

We used the latest round of the India Human Development Survey-II (IHDS-II), 2011–12 for our analysis. IHDS-II is a nationally representative, multi-topic survey of 42,152 households in 1,503 villages and 971 urban neighbourhoods across India. The survey collects a wide range of information on household health, education, employment, economic status, marriage, fertility, gender relations, social capital, village infrastructure, wage levels, and panchayat composition. IHDS-II is the most up-to-date household survey available on India. Also, it contains a comprehensive set of information on gender relations and women status in the household such as their autonomy, ability, freedom, exposure to information and bargaining power that allows us the unique opportunity to study the relationship between these variables and child birth registration in India.

Our sample contains information on 9333 children less than 5 years old in 31 states, 367 districts and 2189 villages/neighborhoods. India is a large country with 31 states and 5 union territories. Those are subdivided into 686 districts and districts into a few more layers of administrative units. The Maternal and Child Health (MCH) care is implemented through the Department of Family Welfare (DFW) mostly at the district and sub-district levels through different levels of health care delivery systems e.g. Subcentres (SCs), Primary Health Centres (PHCs), Community Health Centres (CHCs) and District Hospitals. Previous studies have found significant disparities in MCH service coverage and efficiency differences in service delivery across districts in India [19, 20]. Therefore, along with individual and household level characteristics, we have considered villages/neighborhoods and districts as the other two higher levels of analysis in a mixed effects hierarchical model as policies and service provisions at these levels might influence birth registration. We have included state level covariates at the individual level analysis.

### 2.2 Methodology

We use multilevel models to take account of the hierarchical or clustered structure of the data. For example, children who live in the same household are more likely to have similar outcomes for birth registration than children randomly chosen from the population at large. Households are further nested within communities with children living in the same communities facing the same set of cultural and institutional barriers and enabling factors for birth registration than those living in other communities. Communities are also nested within higher administrative units such as districts with children living in the same districts likely to share similar policy and health care infrastructure than those living in other districts. However, our analysis does not consider multiple children from the same household because the birth registration information in the data pertains to the mother’s last birth only [21–24].

Our dependent variable in the study is a binary response variable with a value 1 ‘if the child has a birth certificate’ and 0 otherwise. It is based on the survey response to the question ‘if the mother possesses a birth certificate for her last birth that occurred in the last 5 years (i.e. since January 2005)’.

Our explanatory variables are grouped into three levels to reflect the hierarchical nature of the data. Level 1 variables correspond to child/household/maternal characteristics with community and state level contextual covariates. Level 2 variables correspond to community as a random effect and community/village level characteristics as random slopes and Level 3 corresponds to district as a random effect and some of child level maternal characteristics as random slopes. We run a three level mixed effects random slope logit model.

We started our estimation by running two mixed effects logit null models with no covariates [24]. The first null model introduces a random intercept component at Level 3 (district level) while the second introduces an additional random intercept term at Level 2 (community level). Then, we introduce state, community, maternal, household and child level covariates with one slope coefficient at Level 2 and two slope coefficients at Level 3 in a step wise manner following forward selection in a three level mixed effects random slope logit model. The aim here is to study any variations in the null models that were due to each of the confounding factors.

The corresponding equations for the two null and full mixed effect models are presented below [24]. The first null model, a mixed effects binary response logit model with a random intercept component at the Level 3 (district level) can be represented as

$${\mathrm{logit}\left(y_{ik}=1\right)=\beta }_{0}+v_{0k}$$ (1)

$$v_{k}\sim N(0,\sigma^{2})$$

The latent variable formulation is

$${y_{ik}^{*}=\beta }_{0}+v_{0k}+e_{ik}$$ (2)

Where, *y*_*ik*_ is the outcome variable for whether the i^th^ child in the k^th^ district has a birth certificate, *β*_0_ the overall sample mean, *v*_0*k*_ the district level random intercept–it is the effect of being in district k on the log-odds that y = 1, *σ*^2^ the district level (residual) variance, or the between-district variance on the log-odds that y = 1, and *e*_*ik*_ the individual level residuals. In a two-level model the aim is to split the residual variance into two components corresponding to the two levels in the data structure [24]. The second null model with an additional Level 2 (community) random intercept, *u*_0*j*_ within districts can be represented as

$${logit(y}_{ijk}=1)=\beta_{0}+v_{0k}+u_{0jk}$$ (3)

$$v_{k}\sim N(0,\sigma_{v}^{2})\;and\;u_{jk}\sim N(0,\sigma_{u}^{2})$$

Finally, we model the binary response for whether the child has a birth certificate or not as a three level logistic random slope model that can be represented as

$$logit(y_{ijk}=1|x_{ijk},u_{jk},v_{k})=\beta_{0}+\beta_{1}x_{1ijk}+\beta_{2}{\;x}_{2ijk}+\beta_{3}\;x_{3jk}+\beta_{4}\;x_{4k}+v_{0k}+v_{1k}\;x_{1ijk}+v_{2k}\;x_{2ijk}+u_{0jk}+u_{1jk}\;x_{3jk}$$ (4)

Where *u*_0_ and *u*_1_ are the random intercept and slope coefficients at the Level 2 (community level) that are assumed to follow normal distributions with zero means, variances $\sigma_{u0}^{2}$ and $\sigma_{u1}^{2}$ respectively, and covariance *σ*_*u*01_. Because *u*_0*jk*_ and *u*_1*jk*_ are allowed to be correlated (i.e. *σ*_*u*01_ is not assumed to equal zero), they are expected to follow a *bivariate normal* distribution that can be represented as

$$\left(\begin{array}{l} u_{0}\\ u_{1} \end{array}\right)=\left(\left(\begin{array}{l} 0\\ 0 \end{array}\right),\left(\begin{array}{ll} \sigma_{u0}^{2} & \sigma_{u01}\\ \sigma_{u01} & \sigma_{u1}^{2} \end{array}\right)\right)$$

Similarly, *v*_0_ is the random intercept and *v*_1_, and *v*_2_ are the random slope coefficients at the Level 3 (district level) that can be represented as

$$\left(\begin{array}{l} v_{0}\\ v_{1}\\ v_{2} \end{array}\right)\sim N\left(\left(\begin{array}{l} 0\\ 0\\ 0 \end{array}\right),\left(\begin{array}{lll} \sigma_{v0}^{2} & \sigma_{v01} & \sigma_{v02}\\ \sigma_{v01} & \sigma_{v1}^{2} & \sigma_{v12}\\ \sigma_{v02} & \sigma_{v12} & \sigma_{v2}^{2} \end{array}\right)\right)$$

All analyses were conducted using STATA-14. We calculated robust standard errors that are clustered by districts to relax the assumption of independent and identically distributed errors within districts.

### 2.3 Explanatory variables

This research is conceptually aligned to the literature on maternal and child health (MCH) which focuses on factors influencing the utilisation of maternal and new born health services (in low and middle-income countries). The factors associated with the utilisation of MCH related services (such as prenatal, delivery and postnatal services) are unlikely to be different from those that influence birth registration decision in the period following the birth of a child. The literature identifies a number of factors that are associated with the utilisation of such services including: lack of education (i.e., mother’s and husband’s education level); lack of decision-making authority (i.e., women’s authority and autonomy); socio-economic barriers (i.e., low household living standards, low household income, no insurance coverage); social class structure and religion (i.e., religion and caste of the household); limited access to healthcare facilities (i.e., transportation); geographical location (i.e., distance to health care facilities); and lack or shortage of trained and skilled health care professionals (i.e., capacity and knowledge of skilled health care professionals) [25].

Some recent studies on determinants of birth registration have highlighted the association between child, household and community level sociodemographic and economic factors and birth registration. Amo-Adjei and Annim (2015) [26] find that mother’s education, household wealth and urban residence are positively associated with the likelihood that a child is registered in Ghana. Religion is also found to be a significant determinant of birth registration with children whose parents practice a traditional religion at a significant risk of not being registered. There is also evidence of significant regional effect with children from eastern region of Ghana less likely to be registered [26]. Okunlola et al. (2017) [27] similarly find that birth registration increases with household wealth index and educational attainment of the mother in Nigeria. It is also noted that lack of access to registration services and indirect costs associated with registration contribute to low birth registration. According to Chereni (2016) [28], social and cultural factors are equally important in influencing birth registration as economic factors in Zimbabwe. And birth registration is viewed as an outcome that results from the interaction between economic, non-economic, personal and structural factors. Isare and Atimati (2015) [29] advocate for a community based approach where birth registration centers are established within communities to increase accessibility and awareness about the benefits of birth registration. Our study controls for a comprehensive list of variables associated with birth registration in the wider literature and in India (in particular) [25].

As noted above, our explanatory variables are grouped into three levels. The variables at Level 1 correspond to child/household/maternal characteristics with village/community level and state level contextual covariates. The child level attributes include—child’s age as a continuous variable and dummy variables representing institutional/non-institutional place of birth and gender of the child. Parental level attributes included are—mother’s age and educational level of both parents as continuous variables, and categorical variables representing mother’s self-assessed health status. Mother’s migrant status representing mother’s childhood place of residence (i.e. if the mother’s natal family resides in the same village/town or another) and mother’s Ante Natal Care (ANC) seeking behavior, in particular, if the mother had four or more ANC visits during her last pregnancy are also included. We have also included a variable representing the proportion of children who have died out of the total children ever born to the mother at the time of the survey.

More importantly, we have included a wide range of variables representing gender relations and mother’s social and economic status in the household such as mother’s role in household decision making, her control over household resources, her ability to independently visit places of need, her freedom of movement and bargaining capacity in the household. Understanding how women’s autonomy, ability and freedom at the household level are associated with birth registration and how factors at the community or higher sociopolitical level (such as districts) moderate this relationship is an important contribution of this research. Previous studies have highlighted the positive influence of maternal autonomy on feeding practice, birth weight and infant growth in India [30 –32]. It is also noted in the literature that maternal autonomy could increase self-motivation and bring about behavioral change that would improve the welfare of the mother and her family. A systematic review by Upadhyay et al. (2014) [33] finds a positive association between women’s empowerment and lower fertility, longer birth intervals, and lower rates of unintended pregnancy.

Methodologically, Upadhyay et al. (2014) [33] highlighted the importance of choosing appropriate measures that better approximate women’s empowerment. It was further noted that studies that used multiple and multidimensional measures of empowerment were more likely to find consistent results. Shroff et al. (2011) [31] conducted a confirmatory factor analysis to develop multiple dimensions of maternal autonomy. They find that individual dimensions of autonomy could operate differently to influence child growth and wellbeing. Accordingly, we identified 20 variables in our dataset that described mother’s autonomy, ability, freedom, exposure to information/other resources and bargaining power in the household [32, 34, 35] and conducted factor analysis to summarize and identify any common underlying theme. Our analysis clearly identified four different underlying constructs or factors in these variables and depending on the nature and category of the variables clubbing under each of these factors we have named Factor 1 as ‘Mother’s Autonomy’, Factor 2 as ‘Mother’s Ability’, Factor 3 as ‘Mother’s Freedom in Movement’ and Factor 4 as Mother’s Bargaining Capacity. However, three variables that we identify as mother’s exposure to outside world did not group into any of these four factors and we have decided to include them independently in the model. The set of variables grouped under each factor, their rotated factor loadings (pattern matrix), unique variances and the three independent mother’s exposure variables are listed in Table 1 below.

**Table 1:** Mother’s autonomy, ability and freedom: Rotated factor loadings (pattern matrix) and unique variances.

Variables (No. of Observations = 9995)	Factor 1: Mother’s Autonomy	Factor 2: Mother’s Ability	Factor 3: Mother’s Freedom in Movement	Factor 4: Mother’s Bargaining capacity	Unique Variation in Variables
**1. Mother has most say or decides jointly on what to cook on a daily basis**	0.851	0.0869	0.0326	0.0981	0.2576
**2. Mother has most say or decides jointly on purchasing expensive item**	0.9482	0.0326	-0.0418	-0.0219	0.0977
**3. Mother has most say or decides on number of children she has**	0.8962	0.0837	-0.103	0.0085	0.1791
**4. Mother has most say or decides what to do if she falls sick**	0.8929	0.0663	-0.0072	0.1165	0.1847
**5. Mother has most say or decides jointly on whether to buy land/property**	0.9677	0.0101	-0.0467	-0.0222	0.0608
**6. Mother has most say or decides jointly on wedding expense**	0.9532	0.0481	-0.0091	-0.0265	0.0883
**7. Mother has most say or decides jointly on what to do if a child falls sick**	0.9404	0.0986	0.1131	0.0745	0.0875
**8. Mother has most say or decides jointly to whom her children should marry**	0.9256	0.0885	0.0533	0.0358	0.1313
**9. Mother had jointly/solely chosen her husband in marriage**	0.0681	0.8948	0.1525	-0.0206	0.1711
**10. Mother’s natal family status same/better than in-laws**	0.1297	0.8959	0.2178	0.021	0.1327
**11. Mother can go short distance by train/bus alone**	0.0476	0.8982	0.281	0.0171	0.1117
**12. Mother can visit Health Centre alone**	0.1009	0.8513	0.1756	0.0244	0.2336
**13. Mother can visit relative/friend alone**	0.0131	0.2701	0.9266	-0.0257	0.0676
**14. Mother can visit local shop alone**	-0.049	0.2277	0.8968	0.0199	0.1412
**15. Mother does not need permission/must inform to visit Health Centre**	0.0043	0.1846	0.9249	-0.0123	0.1102
**16. Mother does not need permission/must inform to visit relative/friend**	0.1919	-0.1637	0.0559	0.4965	0.6867
**17. Mother does not need permission/must inform to go short distances by train/bus**	0.0446	0.0636	-0.0347	0.8653	0.244

Note: The three independent mother’s exposure variables are–(1) Mother’s been to a metropolitan city/another state/abroad in last 5 years; (2) Family outings to cinema, fair or restaurant; (3) Mother often discusses with husband about work/farm/expenditures/community/politics. We have used Factor analysis with method: principal-component factors, rotation: orthogonal varimax (Kaiser off) and have retained 4 factors. The overall Kaiser-Meyer-Olkin measure of sampling adequacy, KMO is 0.6979.

The household level variables that we control for include urban-rural residence, household wealth status captured using wealth quintiles, caste and religious affiliation of the household. We also control for state level contextual covariates in our regression:—health expenditure as a percentage of Net State Domestic Product (NSDP), per capita public expenditure on health, literacy rate, gross enrolment rate, infant mortality rate and a dummy variable identifying the low-income states in India. The state level variables were all extracted from the *Economic Survey* 2012–13 [36] and publication from National Health Accounts Cell [37].

At Level 2, we have controlled for the community/village level random effects and a random slope for proportion of institutional births in the village. Additionally, village/community level variables are included as Level 1 contextual covariates in the model. These include village/community level mean years of schooling, proportion of institutional births and quintiles of median per capita household consumption expenditures. Given that IHDS-II used cluster sampling, all community level variables were created by aggregating relevant individual survey responses at the clusters.

At Level 3, we have controlled for the district level random effects and random slopes for two maternal socio-economic status summary variables representing Mother’s Autonomy and Ability. Previous studies have highlighted the need to account for the influence of communities and broader socio-political environment on women’s empowerment. It is documented that an individual woman’s empowerment process is simultaneously shaped by individual, social, cultural and political forces [34]. This calls for multilevel modelling to analyse the complex interactions between women empowerment measures at individual and at higher than the individual level [33, 38]. All continuous variables in our model are centered at their mean values.

### 2.4 Descriptive statistics

A summary statistics of the variables is presented in Table 2. Table 2 shows that over 62 per cent of children in our sample have birth certificates. The average age of children is 2.19 years and 55 percent are male. Also, over 71 percent of children in the sample were born in an institution.

**Table 2:** Descriptive statistics.

		Descriptive statistics (Sample size = 9333)		
	Mean/proportion	Standard Deviation	Min	Max
***State level factors***				
Literacy rate (%)	74.54	6.79	63.82	93.91
Gross Enrolment rate (%)	104.4	10.34	78.2	155
Health expenditure as % of NSDP	1.07	0.47	0.54	4.79
Per capita public expenditure on health	363.28	363.28	298.43	3719.77
Infant mortality rate (%)	42.26	12.90	11	59
Low income status (%)				
Low income states	54.0	0.50	0	1
Non-low income states				
***Community level factors***				
Quintile of Cluster median Per capita consumption expenditure	3.25	1.39	1	5
Cluster mean years of schooling	2.98	0.97	0	9
Cluster proportion of institutional delivery (%)	70.32	0.29	0	100
***Household and mother level factors***				
Mother’s age (in years)	27.96	5.45	15	60
Mothers education	6.09	5.01	0	16
Fathers education	7.53	4.70	0	16
Mother’s self-assessed health status				
Very good				
Good	53.05	0.50	0	1
Ok	14.56	0.35	0	1
Poor and very poor	5.13	0.22	0	1
Mother’s place of childhood residence				
Same village or town				
Another village	65.77	0.47	0	1
Another town or metro city	17.85	0.38	0	1
Mother’s number of Antenatal (ANC) Check-ups at last pregnancy		0.68		
No ANC Check-ups	11.87	0.32	0	1
Up to 3 ANC Check-ups	42.71	0.49	0	1
4 and above ANC Check-ups	45.42	0.50	0	1
Proportion of children who have died (out of those ever born to mother)	3.71	0.11	0	0.833
Household wealth quintile				
First quintile	15.57	0.36	0	1
Second quintile	17.98	0.38	0	1
Third quintile	20.53	0.40	0	1
Fourth quintile	22.50	0.42	0	1
Fifth quintile	23.42	0.42	0	1
Religious background of the household				
Hindus				
Muslims	15.76	0.47	0	1
Others	3.10	0.17	0	1
Caste				
Brahmin and other forward				
Other backward castes	40.72	0.49	0	1
Scheduled castes and tribes (SC & ST)	32.69	0.47	0	1
Others	1.14	0.11	0	1
Place of Residence (%)				
Metro urban				
Other urban	23.77	0.43	0	1
More developed villages	29.42	0.45	0	1
Less developed villages	40.81	0.49	0	1
Household Size	6.30	2.58	2	30
***Child-specific factors***				
Age of the child (in years)	2.19	1.65	0	5
Sex of child				
Male	54.86	0.50	0	1
Female				
Place of Birth of child				
Institutional delivery	71.38	0.45	0	1
Non-institutional delivery				
***Mother’s social and economic status indicators***				
Mother’s autonomy	0.79	0.31	-0.16	1.12
Mother’s ability	0.67	0.44	-0.43	1.26
Mother’s freedom of movement	0.00	0.33	-0.39	1.23
Mother’s bargaining capacity	0.98	0.49	-0.26	2.35
Mothers who have been to a metro/another state/abroad in past five years	29.00	0.45	0	1
Mothers who have gone out on family outings to cinema, restaurants etc	61.63	0.49	0	1
Mothers who discuss with husband different issues including politics, work etc	57.49	0.49	0	1

Mothers’ characteristics reveal that the average mother is 28 years of age and had received around 6 years of schooling. Moreover, over 80 percent of mothers self-assess their health status as good or very good. Only 45 percent of mothers have received the recommended 4 and above ANC check-ups during their last pregnancy. The summary statistics of the mother’s socio-economic status related factor variables–namely, mother’s autonomy, mother’s ability, mother’s freedom of movement and mother’s bargaining capacity are presented in the table. In addition, the variables representing mother’s exposure to the outside world reveal 29 per cent of mothers in the sample have been to a metropolitan city, another state or abroad in the past five years. In contrast, a larger proportion—about 62 percent—state that they have gone out on family outings while 57 percent maintain that they often engage in discussions with their husbands on various topics including work, community and politics.

Table 2 also presents that around 81 percent of households in the sample are Hindus. In terms of caste affiliation, ‘other backward castes’ make up 41 percent of our sample while ‘Scheduled castes and tribes’ account for 33 percent and ‘Brahmin and other forward’ castes 25 percent. Only 6 percent of sampled households reside in ‘metropolitan urban areas’ with 41 percent coming from ‘less developed villages’, 29 percent from ‘more developed villages’ and 24 percent from ‘other urban areas’.

## 3. Discussion of results

**Fig 1:**
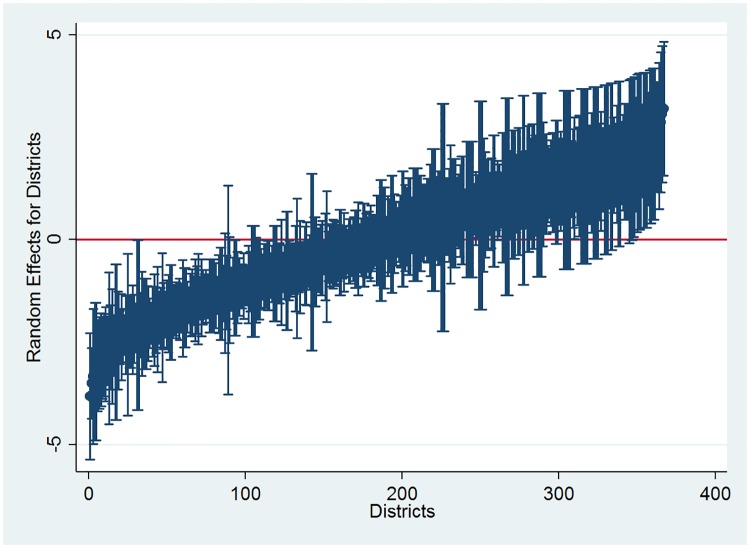
District level differences in birth registration coverage.

We have estimated a couple of null models in the beginning. The first null model is estimated with only district-level random effects and the second null model with both district-level and village/community level random effects. The results from these models are presented in Table 3. The intraclass correlation coefficient (ICC) in model 1 indicates that 46.7 percent of the total variation in birth registration in India lies between districts while the remaining 53.3 percent lies within-districts. Fig 1 below shows a caterpillar plot of the residuals for all 367 districts in the sample from model 1 together with 95% confidence intervals. For a substantial number of districts, the 95% confidence interval does not overlap with the horizontal line at zero, indicating that birth registration coverage in these districts is significantly above average (above the zero line) or below average (below the zero line) [Also see 24].

**Table 3:** Null models: With District Effects (Model 1) and District and Community Effects (Model 2).

	Model 1 (95% CI)	Model 2 (95% CI)
**Intercept**	0.937 (0.744, 1.130)	1.071(0.859, 1.283)
**Between District variance**	2.885 (2.366, 3.517)	3.266 (2.651, 4.023)
**Between Community variance**	-	0.834 (0.654, 1.065)
**ICC (District)**	0.467 (0.418, 0.517)	0.442 (0.392, 0.494)
**ICC (District and Community)**	-	0.555 (0.510, 0.599)

As within-district differences account for most of the variation in birth registration, we looked at the effect of village/community level differences within districts on birth registration in model 2. The ICC from model 2 indicates that 44 percent of the variation in birth registration is explained by between district differences while between community differences account for 11 percent and within community differences for 45 percent of the variation.

As noted above, within-community differences [i.e. differences at the household and individual level] account for most of the variation in birth registration within-districts. We, therefore, estimate a comprehensive model which controls for household and individual child level covariates at level 1 including mother’s social and economic status, state and community level contextual characteristics. We also allow for two random slopes at the district level for mother’s social and economic status indicators–namely, mother’s autonomy and mother’s ability. A random slope at the community level is also included for ‘proportion of institutional delivery at the village level’. The results from estimation of this model are shown in Table 4.

**Table 4:** The three levels random slope model.

	Odds Ratio	Standard Error	95% Confidence Interval	
			**Lower**	**Upper**
***State level factors***				
Literacy rate (%)	1.000	0.019	0.963	1.039
Gross Enrolment rate (%)	1.024**	0.009	1.006	1.042
Health expenditure as % of NSDP	0.487***	0.112	0.310	0.765
Per capita public expenditure on health	1.000	0.000	0.999	1.000
Infant mortality rate (%)	0.998	0.010	0.978	1.018
Low Income status				
Low income states	Reference	Reference	Reference	Reference
Non-low Income States	9.157***	2.489	5.375	15.601
***Community level factors***				
Cluster median Per capita consumption expenditure	0.945	0.043	0.865	1.032
Cluster mean years of schooling	1.096*	0.054	0.995	1.206
Cluster proportion of institutional delivery (%)	1.456*	0.284	0.994	2.133
***Household and mother level factors***				
Mother’s age (in years)	1.000	0.007	0.987	1.014
Mothers education	1.045***	0.010	1.025	1.066
Fathers education	1.030***	0.009	1.012	1.049
Mother’s self-assessed health status				
Very good	Reference	Reference	Reference	Reference
Good	0.801***	0.071	0.673	0.954
Ok	0.836	0.099	0.664	1.054
Poor or very poor	0.795	0.124	0.585	1.080
Mother’s place of childhood residence				
Same village or town	Reference	Reference	Reference	Reference
Another village	0.989	0.101	0.810	1.208
Another town or metro city	1.049	0.129	0.824	1.335
Mother’s number of antenatal visits at last pregnancy				
No ANC Check-ups	Reference	Reference	Reference	Reference
Up to 3 ANC Check-ups	1.756***	0.197	1.409	2.188
4 and above ANC Check-ups	2.180***	0.272	1.707	2.783
Proportion of children who have died (out of those ever born to mother)	0.891	0.245	0.519	1.529
Household wealth quintile				
First quintile	Reference	Reference	Reference	Reference
Second quintile	1.289**	0.140	1.043	1.594
third quintile	1.261**	0.142	1.012	1.572
Fourth quintile	1.329**	0.161	1.048	1.685
Fifth quintile	1.496***	0.205	1.143	1.958
Religious background of the household				
Hindus	Reference	Reference	Reference	Reference
Muslims	0.804*	0.092	0.643	1.006
Others	1.032	0.237	0.658	1.619
Caste				
Brahmin and other forward	Reference	Reference	Reference	Reference
Other backward castes	0.953	0.091	0.791	1.148
Scheduled castes and tribes (SC & ST)	0.750***	0.076	0.615	0.916
Others	0.861	0.302	0.433	1.712
Place of Residence (%)				
Metro urban	Reference	Reference	Reference	Reference
Other urban	1.417	0.460	0.750	2.679
More developed villages	1.011	0.329	0.535	1.913
Less developed villages	0.852	0.279	0.448	1.619
Household size	0.986	0.013	0.961	1.011
***Child-specific factors***:				
Age of the child (in years)	1.046**	0.023	1.002	1.091
Sex of child				
Female	Reference	Reference	Reference	Reference
Male	1.010	0.064	0.892	1.144
Place of Birth of child				
Non-institutional delivery	Reference	Reference	Reference	Reference
Institutional delivery	4.602***	0.402	3.879	5.461
***Mother’s social and economic status indicators***				
Mother’s autonomy	1.159	0.157	0.889	1.511
Mother’s ability	1.274**	0.135	1.035	1.568
Mother’s freedom of movement	1.137	0.139	0.895	1.443
Mother’s bargaining capacity	0.907	0.061	0.795	1.035
Mothers who have been to a metro/another state/abroad in past five years	1.181**	0.097	1.007	1.387
Mothers who have gone out on family outings to cinema, restaurants etc	1.144*	0.084	0.991	1.321
Mothers who discuss with husband different issues including politics, work etc	0.920	0.068	0.796	1.064
	**Estimate**	**Standard Error**	**Lower**	**Upper**
***District Level Random Slopes***				
Variance (Mother’s autonomy)	0.776**	0.364	0.310	1.948
Variance (Mother’s ability)	0.679**	0.220	0.359	1.283
Variance (Constant)	1.243	0.422	0.639	2.417
Covariance (mother’s autonomy, mother’s ability)	0.001	0.208	-0.406	0.408
Covariance (constant, mother’s autonomy)	-0.174	0.312	-0.786	0.438
Covariance (constant, mother’s ability)	-0.503**	0.250	-0.992	-0.013
***Community Level Random Slope***				
Variance (Cluster proportion of institutional delivery)	1.37	1.094	0.285	6.559
Variance (constant)	0.552	0.371	0.148	2.064
Covariance (constant, Cluster proportion of institutional delivery)	-0.541	0.649	-1.815	0.732
Number of observations	9,333			
Number of Districts	367			
Number of Communities	2,198			
Log pseudolikelihood	-4036.3502			
Wald χ2 (43)	1026.96			

Note:

*** p<0.01;

** p<0.05;

* p<0.1.

Our primary focus of analysis in this paper is the variables representing mother’s social and economic status and bargaining power in the household. The summary variable (estimated using factor analysis) representing ‘mother’s ability’ is significantly associated with birth registration. Mothers that are able to visit health centres, friends/relatives, a local shop or travel short distance by train/bus on their own are more likely to have their children registered. This variable reflects the mother’s capacity to leave the house unaccompanied and move around without needing a chaperon. Higher maternal mobility is related to greater decision-making ability within the household [39]. As primary caregivers for children, mothers’ ability to move around is crucial for a number of activities that enhance the welfare of children such as immunisation, health check-ups, and possibly birth registration [40]. A mother who is capable of moving around on her own can also register the birth of her children without depending on the husband. We can also see that the random slope coefficient for ‘mother’s ability’ [included at the district level] is statistically significant. This shows that the marginal effect of ‘mother’s ability’ on birth registration is not constant but varies across Indian districts. Moreover, the negative covariance estimate between the intercept and mother’s ability variable indicates that districts with above average birth registration tend to have a flatter than average slope or below average effects of maternal ability.

We also find that two other variables which represent mother’s exposure to outside world–namely, whether the mother had been to a metro/another state/abroad in the last 5 years; and whether she had gone out on family outings- are significant determinants of birth registration. In particular, having a mother who had been to a metro/another state/abroad increases the odds of birth registration by a factor of 1.18. A mother travelling to another state or abroad signifies [that she enjoys] more autonomy in the household [39]. And undertaking such travels present the opportunity to interact with various people and come across different ideas that could enhance the wellbeing of children. Similarly, having a mother that had gone out on family outings to cinemas or restaurants increases the odds of birth registration by a factor of 1.14, which is probably reflecting an already empowered mother with a relatively higher bargaining power in the household or ability to positively influence decisions concerning children [including that of birth registration].

Additionally, we had included a random slope coefficient for ‘mother’s autonomy’ at the district level. The ‘mother’s autonomy’ variable shows mother’s decision-making power on issues such as purchase of property/land, wedding expenses, what to do if a child falls sick and whom the child should marry. A mother that enjoys a greater degree of ‘autonomy’ would have greater access and control over economic resources in the household. It is documented in the literature that autonomous mothers would allocate more resources towards their children [41, 42]. Thus, mothers with control over resources would be more likely to register their children by bearing the direct and indirect costs that might be involved in the birth registration process. The random slope coefficient for this maternal autonomy variable at district level has come out as statistically significant indicating that the marginal effect of this variable significantly varies across districts. It may be reflecting the extent of regional diversity in gender relations in India. Previous studies have shown significant spatial and socio-cultural differences in various dimensions of women’s empowerment across regions in India [43]. For example, it has been argued that women in South India have more voice in family life, more freedom of movement and exposure to the outside world than their counterparts in North India [44]. Gutpa and Yesudian (2006) [43], on the other hand, found that states situated in central part of India (such as Uttar Pradesh, Rajasthan, Madhya Pradesh, Bihar and Orissa) had low empowerment of women. Given the spatial (and socio-cultural) differences in India, our findings highlight that policy initiatives to increase birth registration (or even women’s autonomy for that matter) should be designed by taking district (regional) idiosyncrasies into account.

Among the child specific factors included in the regression, place of birth of child is statistically significant. The odds of birth registration increase by a factor of 4.6 for a child who has had an institutional birth. We can also see that institutional delivery has one of the largest impacts on the likelihood of birth registration in our model. The high likelihood of registration for institutionally delivered children can be attributed to the fact that the reporting of such births to the ‘Registrar of Births and Deaths’ is the responsibility of the medical officer in charge. More importantly, medical officers who attend the birth of a child are obligated to report the incidence to the Registrar [45]. Brito et.al (2013) [46] also find high probability of registration for institutionally delivered children in Latin America and the Caribbean. Our results do not show any significant difference in the odds of birth registration between boys and girls. A multivariate analysis by UNICEF (2005) [47] identifying the determinants of birth registration across 63 countries also concluded that gender is insignificant. Age of child is another significant determinant with the odds of registration increasing with age. Turning to the household and other mother level factors in our model, we see that parental education is positively associated with the likelihood of birth registration. An increase in the mother’s (father’s) years of schooling by one year raises the odds that a child will get registered by a factor of 1.05 (1.03). The positive association between maternal education and birth registration had also been established in other countries [Also see 26, 46]. Harding et.al (2015) [48] investigate the transmission channels through which maternal education affects child outcomes, in particular their academic achievement. One channel explored is social capital with educated mothers more likely to be part of a social network of other educated people who possess the knowledge, skills and resources beneficial for children. Thus, educated mothers can be expected to receive valuable information and advice on various aspects of a child’s life [including the benefits of birth registration]. The positive effect of father’s education may also be a result of similar benefits from father’s social capital.

Mother’s number of antenatal visits at last pregnancy is another significant variable positively associated with birth registration. The odds of birth registration for mothers who have had four or more antenatal check-ups is 2.18 times that of mothers who have had no check-up. Antenatal care provides pregnant women with education, counselling, screening and treatment to ensure mother and foetus remain in good health [49]. Moreover, antenatal visits would increase the mother’s awareness of what to expect after the birth of a child by providing information on postpartum care, including breastfeeding, immunisation, and (quite possibly) the importance of birth registration [50].

Our results also indicate that household wealth is significantly associated with birth registration. The odds of birth registration for households in the top wealth quintile is 1.49 times that of households in the bottom quintile. Other cross-country studies have also established the positive impact of wealth status on birth registration [2, 47]. This might be capturing the possibility that households in lower wealth quintiles are put off birth registration by the late fee that applies for children not registered within 21 days of birth. Office of the Registrar General (2010) [45] also notes that births not registered after 30 days but within a year can be registered on production of an affidavit and permission from the prescribed authority on top of the prescribed late fee. Amo-Adjei and Annim (2015) [26] had demonstrated that late fee was a barrier for registration in Ghana. There might also be indirect costs such as cost of transportation or income lost due to time away from work which might hinder poorer households from having their children registered [2]. Also, it might simply be the fact that poor households fail to realise the long term benefits of registering their child birth.

Moreover, the dummy variable for religious background shows that the odds of birth registration for a child born in a Muslim family is 0.80 times that of a child born in a Hindu family. The public health literature in India which has examined the Hindu-Muslim differences in fertility planning may shed some light into this result. There are studies that indicate higher level of unmet need for family planning among Muslims [51]. Singh et. al (2012) [52] also find that utilisation of safe delivery care was significantly lower among Muslim women than women from other religions in India. It has also been pointed out that mistrust of government family planning programs and clinics may prevent Muslims from availing themselves of family planning services [51]. In another context, Hussain et. al (2014) [53] attribute the significant Hindu-Muslim disparity in the incidence of Polio to Muslim mistrust of the Polio eradication program in India, which in his view may be rooted in the socio-political and historical context of the country. Similar factors may be at play here making Muslims less likely to register their children than Hindus.

We also find that a child belonging to Scheduled Castes (SC), Scheduled Tribes (ST) or other castes has a lower probability of registration compared to the Forward/General castes. Since India’s independence in 1947 the social caste structure has been identified as a significant hindrance for the socioeconomic development of minority groups. Despite government effort to empower lower caste groups and minorities, significant disparities across all indicators of development continue to exist. Studies indicate that social caste structure in India is a significant predictor for safe delivery and post-natal care utilization with women from Scheduled Castes (SC) and Scheduled Tribes (ST) less likely to access such services [52]. Thus, it is not surprising that we also find women from these minority groups are less likely to register their children given their less inclination to access post-natal and other services. Nevertheless, further research needs to establish whether this is due to some cultural practice or lack of proper knowledge or awareness in these communities. There is similar evidence from Ghana that members of minority ethnic or religious groups are less likely to register compared to majorities [47].

Among the community level factors included in our model, mean years of schooling and proportion of institutional delivery come out as significant. An increase in mean years of schooling by one year is associated with 1.1-fold increase in the odds of birth registration. Thus, education is important not only at the household level but also at the community level. Living in a community with a higher proportion of institutional births also raises the probability of birth registration. These results point out that there are advantages that spill over to the individual/household from living in a community with high mean years of schooling and institutional delivery. We have included a random slope coefficient for proportion of institutional delivery at community level to allow for the effect of this variable (on birth registration) to vary across communities in India but this didn’t come out as significant.

Living in non-low income states has the largest positive effect on birth registration in our regression. The odds of birth registration for a child living in a non-low income state is 9.2 times that of a child living in low income state. This might be reflecting the poor state of health and other infrastructure leading to poor service delivery in low income states in India. Chotia and Rao (2015) [54] note that ‘….low income (BIMARU) states……still lack basic health infrastructure in many of their villages and towns leading to low positions in health index rankings’. The gross enrolment ratio at the state level is also significant and is associated with a higher birth registration. However, health expenditure as percentage of state GDP negatively affects birth registration. This result might be capturing the possibility that national birth certificate campaigns to raise birth registration in India may have largely targeted those states where health expenditure as a percentage of GDP is lower.

## 4. Conclusion

This paper examines the determinants of birth registration in India using a multilevel hierarchical mixed model. In particular, we looked at mother’s autonomy, ability and bargaining power within the household and its significance for child birth registration. The rationale behind using a multilevel mixed effects model is the hierarchical nature of the data in that child and households are nested within communities, which in turn are nested within districts. The estimation results provide us with useful policy implications to increase birth registration in India. Our results show that 45 percent of the variation in birth registration in India lies between individual level differences and 44 percent lies between district level differences and remaining 11 percent lies between community level differences. This brings the policy focus on individuals and districts for targeting birth registration. At individual level the results indicate that the summary variable representing mother’s ability increases the probability of birth registration. Ability to move around independently is an important trait for the mothers because such mothers do not have to wait for their husbands to take their children for immunisation, health check-ups, birth registration…etc. Two variables representing mother’s exposure to outside world [i.e. whether the mother had been to a metro/another state/abroad in the last 5 years; and whether she had gone out on family outings] are also significant. So, mothers that have better bargaining power in the household and are exposed to liberating and progressive ideas from their surrounding that they could use to advance children’s welfare are more likely to register the birth of their children. Again, random slopes at the district level for two indicators of mother’s bargaining power- namely, mother’s ability and mother’s autonomy- came out as significant confirming that while these variables significantly influence birth registration, the level of their influence varies across districts in India. So, policies targeting to empower mothers to improve maternal and child health outcomes and birth registration should have a district level focus.

Our estimates further showed that institutional delivery is a highly significant determinant which increases the probability of birth registration. This is consistent with a priori expectation as medical officers presiding over delivery are duty bound to report the birth. The number of mother’s antenatal visits also came out as significant, which is not surprising given that mothers will also get advice on postpartum care when they access antenatal services. Finlayson and Downe (2013) [55] note that costs of visiting antenatal facilities (even when antenatal services are provided for free) are the major reason behind low access to antenatal care in India (as in other low and middle income countries). The Janani Suraksha Yojana cash transfer program in India, where pregnant women are given a small sum of money to attend antenatal care and deliver in a recognised health care facility has had a significant success in increasing antenatal attendance [55]. Such cash transfer programs with a view to encouraging women’s attendance of antenatal care should be continued and extended to include birth registration given the success of the Janani Suraksha Yojana program.

Robertson (2013) [56] notes that cash transfer programmes are an increasingly popular approach to meet health and development needs of vulnerable children. For example, the conditional cash transfer program rolled out in Mexico under PROGRESA led to increased preventative care, including prenatal care and child nutrition monitoring, and higher school enrolment [57]. In the African context, a social cash transfer program in Malawi reduced child morbidity and increased school enrolment. Similar benefits had also been observed in Kenya and South Africa. More importantly, Robertson et al (2013) [56] found that conditional cash transfers led to increase in the proportion of children with birth certificates in Zimbabwe. Baruah et al (2013) [58] similarly established the positive impact of conditional cash transfer on the birth registration of female children in Assam India. Under the conditional cash transfer scheme, known as Majoni scheme, girls born after February 1, 2009 get 5000 rupees deposited in a bank account given institutional delivery of the female child and compulsory registration among other things. The scheme resulted in an increase in the formal request to have female children registered from 24 percent to 39 percent [58].

Our study also established that probability of birth registration increases by wealth quintile. The direct and indirect costs associated with the process of registration may discourage households in lower wealth quintiles from having children registered. We also find disadvantaged groups such as Scheduled Castes (SC), Scheduled Tribes (ST) or other castes have a lower probability of birth registration compared to the Brahmin and Forward/General castes. Children in low income states are also less likely to be registered. This suggests that policy should give special attention to raising birth registration rates of these disadvantaged groups and states.
